# Anti‐Inflammatory Cytokine Signatures in Primary Glaucoma Subtypes: Diagnostic and Pathophysiological Insights From IL‐36Ra, IL‐37, and IL‐38

**DOI:** 10.1155/joph/1806762

**Published:** 2026-06-23

**Authors:** Yawen Li, Xuanqi Zhang, Xiaowei Yan, Yulei Geng, Kuitang Shi, Weijia Li, Tianyu Zhang, Jiaming Lu, Hengli Zhang, Yizhen Tang

**Affiliations:** ^1^ Department of Ophthalmology, Shijiazhuang People’s Hospital, Shijiazhuang, Hebei, China; ^2^ Center for Quantitative Biology, Academy for Advanced Interdisciplinary Studies, Peking University, Beijing, China, pku.edu.cn; ^3^ Beijing Ophthalmology and Visual Sciences Key Laboratory, Department of Ophthalmology, Beijing Tongren Hospital, Capital Medical University, Beijing, China, ccmu.edu.cn

**Keywords:** aqueous humor, cytokines, interleukin-36 receptor antagonist, interleukin-37, interleukin-38, primary glaucoma

## Abstract

**Objectives:**

To quantify interleukin‐36 receptor antagonist (IL‐36Ra), interleukin‐37 (IL‐37), and interleukin‐38 (IL‐38) levels in aqueous humor and plasma of patients with primary glaucoma, and investigate their correlations with glaucomatous structural damage.

**Methods:**

Matched aqueous humor and plasma samples were collected from patients with acute primary angle‐closure glaucoma (APACG), chronic PACG (CPACG), and primary open‐angle glaucoma (POAG), and age‐related cataract controls (ARC). Levels of IL‐36Ra, IL‐37, and IL‐38 were quantified and correlated with average retinal nerve fiber layer thickness (RNFLT).

**Results:**

A total of 96 participants were enrolled, including 25 APACG, 23 CPACG, 23 POAG, and 25 ARC. In aqueous humor, IL‐36Ra was significantly elevated in CPACG compared to all other groups, while IL‐37 and IL‐38 were markedly increased in both CPACG and POAG compared to ARC and APACG (all *p* < 0.05). In plasma, IL‐36Ra was significantly lower in the POAG and APACG than in ARC and CPACG, and IL‐38 was reduced in APACG compared to all other groups (all *p* < 0.05). All three cytokines in aqueous humor, as well as plasma IL‐36Ra and IL‐38, showed significant negative correlations with average RNFLT. Notably, aqueous humor IL‐38 exhibited the strongest association with RNFLT, particularly within the POAG (*r* = −0.745, *p* < 0.001) and CPACG (*r* = −0.496, *p* = 0.016) subgroups, suggesting an association with structural damage in chronic glaucoma.

**Conclusion:**

Aqueous humor IL‐38 levels correlated with RNFLT thinning in POAG and CPACG, suggesting a potential role as a biomarker associated with structural damage. IL‐36Ra was independently elevated in CPACG, supporting its potential as a subtype‐related biomarker. Further studies are needed to clarify the mechanisms of these cytokines in glaucoma pathogenesis.

**Summary**
• First report of IL‐36Ra/IL‐37/IL‐38 co‐expression in aqueous humor and plasma across primary glaucoma subtypes.
• IL‐36Ra is independently elevated in CPACG, suggesting its potential as a subtype‐related biomarker.
• IL‐38 correlates with RNFLT thinning in chronic glaucoma, suggesting an association with structural damage.

## 1. Introduction

Glaucoma comprises a group of progressive optic neuropathies characterized by the degeneration of retinal ganglion cells (RGCs), optic nerve head cupping, and corresponding visual field (VF) loss. It remains the leading cause of irreversible blindness worldwide. According to recent epidemiological data, the global prevalence of glaucoma exceeded 76 million in 2020 and is projected to rise to 111.8 million by 2040, posing a significant public health challenge, particularly in aging populations [1]. Although elevated intraocular pressure (IOP) is a major risk factor, the underlying mechanisms contributing to optic nerve damage are multifactorial and complex, involving ischemia and hypoxia, mechanical stress, and autoimmune responses [2–5]. Emerging evidence underscores inflammation as a pivotal driver of glaucomatous damage, with microglial activation, cytokine dysregulation, and autoimmune dysregulation contributing to RGC apoptosis and optic nerve degeneration [6–8]. Notably, inflammatory responses exhibit significant differences among various subtypes of primary glaucoma. Studies indicate that acute primary angle‐closure glaucoma (APACG) may present with explosive, transient, and intense inflammatory reactions due to a rapid increase in IOP, whereas primary open‐angle glaucoma (POAG) demonstrates a chronic, low‐grade persistent neuroinflammatory state [9–11]. Chronic PACG (CPACG) demonstrates an intermediate phenotype, combining elements of acute and chronic inflammation with pathological remodeling of the trabecular meshwork (TM) [12]. These differences suggest that identifying inflammation‐specific biomarkers for different subtypes holds important clinical value for the classification of glaucoma subtypes, disease monitoring, and precision therapy.

The interleukin‐1 (IL‐1) family cytokines—IL‐36 receptor antagonist (IL‐36Ra), interleukin‐37 (IL‐37), and interleukin‐38 (IL‐38)—have emerged as critical regulators of inflammation in many diseases, including peripheral nerve injury, Sjögren’s syndrome, inflammatory bowel disease, and chronic asthma [13–16].

Unlike classical proinflammatory IL‐1 members, these cytokines act as negative feedback modulators of innate immunity [17–19]. IL‐36Ra and IL‐38 inhibit IL‐36 receptor signaling, while IL‐37 suppresses proinflammatory cascades via IL‐18Rα/IL‐1R8 binding and intracellular Smad3‐dependent pathways [20–23].

In the field of ophthalmology, researchers have discovered that, elevated levels of IL‐36Ra, IL‐37, and IL‐38 have been reported in the aqueous humor of patients with acute uveitis, where they appear to mitigate excessive inflammatory responses [24, 25]. In the in vitro high osmotic pressure model of human corneal epithelial cells, IL‐36RA and IL‐38 can act as anti‐inflammatory antagonists to inhibit the production of tumor necrosis factor‐alpha (TNF‐α) and IL beta (IL‐1β) induced by IL‐36*α*, demonstrating potential clinical value in the treatment of dry eye syndrome [26]. Furthermore, increased expression of IL‐36, IL‐37, and IL‐38 was observed in the aqueous humor of CPACG, showing a positive correlation with mean deviation of VF (MDVF), suggesting a potential association with glaucoma inflammation and neurodegeneration [25].

Although the association of IL‐36Ra, IL‐37, and IL‐38 with glaucoma has begun to attract attention, a comparative analysis of their levels across the three major primary glaucoma subtypes—APACG, CPACG, and POAG—within a single patient cohort has not been performed. Current literature has primarily focused on their local intraocular concentrations, specifically in the aqueous humor. In contrast, their systemic levels in plasma and the consequent potential of these cytokines to serve as accessible, noninvasive biomarkers for disease monitoring remain largely unexplored. Furthermore, the association between these anti‐inflammatory mediators and quantitative structural parameters of glaucomatous damage, such as retinal nerve fiber layer thickness (RNFLT), has not been reported.

Therefore, the present study aims to quantify the levels of IL‐36Ra, IL‐37, and IL‐38 in both aqueous humor and plasma samples from patients with APACG, CPACG, and POAG, and to investigate their potential correlations with glaucomatous structural damage, thus providing experimental data and theoretical support for the precise diagnosis and management of glaucoma.

## 2. Materials and Methods

### 2.1. Study Participants

This cross‐sectional observational study involving Chinese subjects was conducted in accordance with the principles of the Declaration of Helsinki and was approved by the Ethics Committee of Shijiazhuang People’s Hospital (Approval No. 2022028; approval date: 30 March 2022). All patients gave informed consent and signed informed consent sheet.

A total of 71 patients (71 eyes) with primary glaucoma and 25 patients (25 eyes) with age‐related cataract (ARC) who met the inclusion criteria were enrolled from the Department of Ophthalmology, Shijiazhuang People’s Hospital. According to the diagnostic criteria [15], glaucoma patients were further classified into three groups: APACG, *n* = 25, CPACG, *n* = 23, and POAG, *n* = 23. The ARC patients served as a comparative group (*n* = 25).

Diagnostic criteria for glaucomatous patients were as follows: APACG: (1) IOP > 21 mmHg; (2) symptoms such as headache, visual blurring, vomiting, and nausea; (3) signs including corneal edema, dilated pupil, shallow anterior chamber, keratic precipitates, glaucomatous flecks, and iris atrophy; (4) fellow eye with shallow anterior chamber and narrow angle. CPACG: (1) IOP > 21 mmHg; (2) gonioscopic evidence of peripheral anterior synechiae involving ≥ 180°of the angle; (3) corresponding VF loss or optic nerve damage. POAG: (1) IOP > 21 mmHg; (2) open angle on gonioscopy; and (3) corresponding VF loss or optic nerve damage. ARC was based on the Interpretation of Cataract in Clinical Guidelines for Ophthalmology [27]. Exclusion criteria were listed below: patients with a history of intraocular surgery, aqueous humor collection is less than 50 μL; patients with a history of other ocular diseases (such as iris neovascularization, trauma, tumor, macular degeneration, and uveitis) or systemic diseases (such as diabetes, systemic infection, autoimmune disease, and neurodegeneration).

Demographic information (gender and age) and medical history were collected for all patients.

### 2.2. Examination

All eyes of the subjects underwent a thorough ophthalmic evaluation. Best‐corrected visual acuity (BCVA) was measured using a Snellen chart and converted to the logarithm of the minimum angle of resolution (logMAR) for analysis. IOP was measured with a calibrated Goldmann applanation tonometer. Anterior segment examination was performed using a slit‐lamp microscope. Gonioscopy was conducted with a single‐mirror Goldmann diagnostic lens to assess the anterior chamber angle. RNFLT of patients was measured by Heidelberg Spectralis OCT. Using the built‐in RNFL program around the disc: circular scanning (with the disc as the center, diameter 3.4 mm). VF testing was performed using the Humphrey Field Analyzer 750i (Carl Zeiss Meditec, USA) with the Swedish Interactive Thresholding Algorithm (SITA) standard 24‐2 strategy. Only reliable VFs were included, defined as fixation losses ≤ 20% and false‐positive/negative errors ≤ 15%. MDVF was extracted for analysis. For each patient, three reliable VFs were obtained at baseline to minimize learning effects; unreliable tests were repeated. All examinations were conducted by experienced ophthalmologists or certified technicians.

### 2.3. Sample Collection

Aqueous humor samples were collected using insulin syringe at the very beginning of the surgery before corneal incision was made using insulin syringe at the very beginning of the surgery before corneal incision was made. All patients received routine preoperative antibiotic eye drops and were not administered any corticosteroids or nonsteroidal anti‐inflammatory drugs before aqueous humor collection. The samples were obtained from the anterior chamber as follows: The insulin syringe was used to collect the anterior chamber from 1 mm inside the limbal (about 100–150 μL). Care was taken to avoid touching intraocular tissue and to prevent contamination of aqueous samples with blood. Aqueous humor was collected in sterile Eppendorf tubes and stored at −80°C until analysis. The levels of IL‐36Ra, IL‐37, and IL‐38 in aqueous humor samples were measured using commercial enzyme‐linked immunosorbent assay (ELISA) kits (Shanghai Enzyme‐linked Biological Technology Co., LTD, China). The experimental procedure was conducted according to the instructions from the manufacturer. All measurements were performed in triplicates.

The blood was collected one day prior to the surgery when patients were admitted to the hospital. Blood samples from participants were collected in heparin tubes at least 6 hours after the last meal. The tubes were immediately transported on ice and immediately processed for centrifugation for 10 min at 3000 g at 4°C. Then the supernatant was collected and immediately stored at −80°C until analysis.

### 2.4. Statistical Analysis

SPSS 27.0 (IBM, IL, USA) statistical software was used for data analysis and to generate the scatter plots for cytokines. The outlier was removed. The data were expressed as mean ± sd. A one‐way ANOVA or Kruskal–Wallis test was used to evaluate differences in the continuous data, including age, cytokine concentrations, average RNFLT, and the post hoc LSD method was used for pair‐to‐group comparison. The chi‐square test was used to assess differences between groups of categorical data such as gender. Pearson correlation analysis was used to analyze the correlation between the levels of IL‐36Ra, IL‐37, and IL‐38 in aqueous humor and the average RNFLT. The receiver operating characteristic (ROC) curve was generated using logistic regression with 10‐fold cross‐validation. To address potential confounding by disease severity, multivariable linear regression analyses were performed with adjustment for age, sex, and RNFLT (or MDVF in sensitivity analyses), using the ARC group as the reference. *p* < 0.05 was considered statistically significant.

## 3. Results

### 3.1. Demographic and Ophthalmologic Characteristics of the Participates

The demographic and clinical data of the subjects, including age, gender, number, and the average RNFLT, are presented in Table 1. A total of 96 participates were recruited including 25 APACG, 23 CPACP, 23 POAG, and 25 ARC. No significant differences in age or gender were observed among groups. Notably, the average RNFLT in APACG is significantly higher than other groups.

**TABLE 1:** Demographic and ophthalmologic characteristics of participates.

	APACG (*n* = 25)	CPACG (*n* = 23)	POAG (*n* = 23)	ARC(*n* = 25)	*F*/*χ* ^2^/*H*	*p*
Age (mean ± sd, years)	68.520 ± 9.803	72.783 ± 7.154	70.826 ± 9.074	69.880 ± 8.917	0.985	0.404^†^
Gender (Male/Female)	6/19	9/14	16/7	18/7	1.384	0.709^§^
With history of smoking, *n*	6	5	7	9		
Disease duration, *n*	0	0	0	0		
BCVA	0.306 ± 0.253	0.366 ± 0.258	0.100 ± 0.133	0.569 ± 0.173	44.592	< 0.001^#^ ^∗^
IOP (mean ± sd, mmHg)	53.600 ± 10.555	34.565 ± 8.938	31.533 ± 6.487	14.680 ± 2.155	76.453	< 0.001^#^ ^∗^
C/D	0.368 ± 0.085	0.704 ± 0.244	0.691 ± 0.271	0.308 ± 0.070	52.723	< 0.001^#^ ^∗^
Glaucoma medications, n						
Prostaglandins	11	11	13	0		
Beta blockers	19	17	16	0		
Alpha agonists	15	13	13	0		
Carbonic anhydrase inhibitors	10	8	11	0		
Average RNFLT (mean ± sd, μm)	110.000 ± 37.340	62.565 ± 15.925	69.000 ± 19.145	91.920 ± 6.570	48.484	< 0.001^#^ ^∗^
MDVF (mean ± sd, dB)	5.825 ± 2.816	13.429 ± 10.154	10.193 ± 7.627	1.091 ± 0.810	49.067	< 0.001^#^ ^∗^

^§^Chi‐square test.

^†^One‐way ANOVA.

^#^Kruskal–Wallis test.

^∗^ *p* < 0.05 was considered significant.

### 3.2. The Level of IL‐36Ra, IL‐37, and IL‐38 in the Aqueous Humor

**FIGURE 1:**
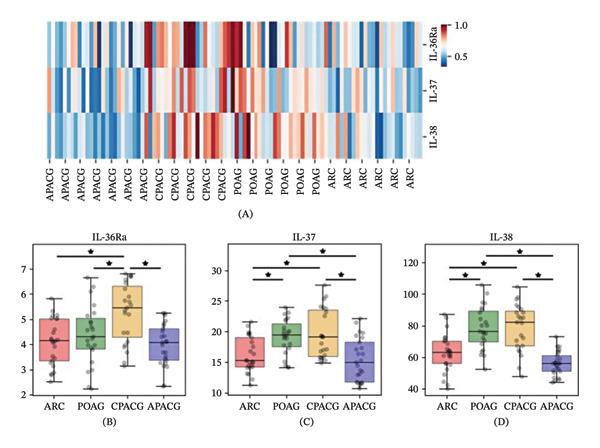
The expression level of IL‐36Ra, IL‐37, and IL‐38 among groups. (A) Heatmap of all cytokines in all groups. (B–D) Group‐wise expression levels of IL‐36Ra (B), IL‐37 (C), and IL‐38 (D) in each group. One‐way ANOVA with post hoc LSD testing were employed to compare aqueous humor levels of IL‐36Ra, IL‐37, and IL‐38. ^∗^ *p* < 0.05 was considered significant.

Aqueous humor samples were collected from participants at the beginning of the surgery. Protein levels of IL‐36Ra, IL‐37, and IL‐38 were quantified, with results presented in Table 2 and Figure 1. Analysis of variance revealed significant alterations in the expression of all three cytokines across disease groups (IL‐36Ra: *F* = 7.70; IL‐37: *F* = 9.32; IL‐38: *F* = 41.88; all *p* < 0.01). Post‐hoc analysis demonstrated significantly elevated IL‐36Ra concentrations in the CPACG group (5.276 pg/mL) compared to the ARC (4.088 pg/mL), APACG (3.978 pg/mL), and POAG (4.378 pg/mL) groups (Table 3). Similarly, IL‐37 concentrations were significantly higher in both the CPACG (19.659 pg/mL) and POAG (19.188 pg/mL) groups compared to the ARC (16.293 pg/mL) and APACG (15.465 pg/mL) groups. IL‐38 levels followed a comparable pattern, with significantly increased concentrations in the CPACG (78.894 pg/mL) and POAG (78.708 pg/mL) groups relative to the ARC (62.760 pg/mL) and APACG (56.035 pg/mL) groups.

**TABLE 2:** Comparison of IL‐36Ra, IL‐37, and IL‐38 levels in aqueous humor.

Cytokine	APACG	CPACG	POAG	ARC	*p*
IL‐36Ra	3.978 ± 0.832	5.276 ± 1.183	4.378 ± 1.174	4.088 ± 0.912	< 0.001^∗^
IL‐37	15.465 ± 3.500	19.659 ± 3.995	19.188 ± 2.885	16.293 ± 2.878	< 0.001^∗^
IL‐38	56.035 ± 7.630	78.894 ± 14.974	78.708 ± 13.684	62.760 ± 12.011	< 0.001^∗^

*Note:* One‐way ANOVA.

^∗^ *p* < 0.05 was considered significant, unit pg/mL.

**TABLE 3:** Comparison of IL‐36Ra, IL‐37, and IL‐38 levels in aqueous humor of patients in each group.

*p* values	IL‐36Ra	IL‐37	IL‐38
APACG vs. POAG	0.183	< 0.001^∗^	< 0.001^∗^
APACG vs. CPACG	< 0.001^∗^	< 0.001^∗^	< 0.001^∗^
APACG vs. ARC	0.706	0.383	0.056
POAG vs. ARC	0.333	0.003^∗^	< 0.001^∗^
CPACG vs. POAG	0.004^∗^	0.634	0.961
CPACG vs. ARC	< 0.001^∗^	< 0.001^∗^	< 0.001^∗^

*Note:* Post hoc LSD method was used for pair‐to‐group comparison.

^∗^ *p* < 0.05 was considered significant.

### 3.3. Correlation Between Average RNFLT and IL‐36Ra, IL‐37, and IL‐38 in Aqueous Humor

**FIGURE 2:**
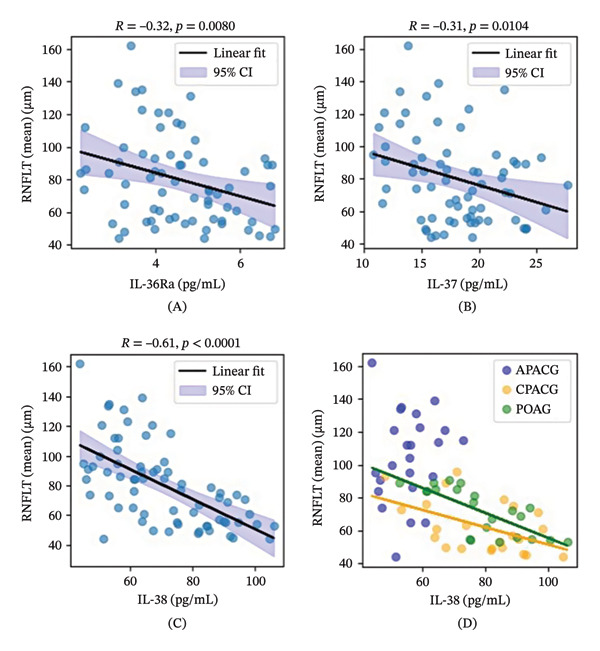
Correlation between average RNFLT and cytokines (IL‐36Ra, IL‐37, and IL‐38) in the aqueous humor. (A–C) Linear regression plots showing the correlation between RNFLT and levels of IL‐36Ra, IL‐37, and IL‐38, respectively. Shaded areas indicate the 95% confidence intervals. (D) Stratified linear regression analysis for CPACG and POAG groups. Blue dots represent APACG, yellow dots represent CPACG, and green dots represent POAG patients.

To assess the clinical relevance of IL‐36Ra, IL‐37, and IL‐38 alterations in the aqueous humor, Pearson correlation analysis was performed between average RNFLT and cytokine concentrations, as shown in Figures 2A–C. All three cytokines exhibited significant negative correlations with average RNFLT. Among them, IL‐38 showed the strongest association (each 1 pg/mL increase in IL‐38 corresponding to an approximate 1.007 μm reduction in average RNFLT).

Next, to further explore this relationship within disease subtypes, subgroup correlation analyses were conducted for the APACG, CPACG, and POAG groups (Table 4 and Figure 2D). No significant correlations were observed between RNFLT and IL‐36Ra or IL‐37 levels within any individual group (*p* > 0.05). However, IL‐38 levels were negatively correlated with average RNFLT in both the CPACG (*r* = −0.496, *p* = 0.016) and POAG (*r* = −0.745, *p* < 0.001) groups, suggesting a subtype‐specific association between IL‐38 and retinal nerve fiber layer thinning.

**TABLE 4:** Pearson correlation analysis of IL‐36Ra, IL‐37, and IL‐38 levels in aqueous humor and mean RNFLT in APACG, CPACG, and POAG groups.

	APACG		CPACG		POAG	
	*r*	*p*	*r*	*p*	*r*	*p*
IL‐36Ra	−0.321	0.118	0.148	0.502	−0.006	0.997
IL‐37	0.161	0.441	0.297	0.169	−0.076	0.729
IL‐38	0.090	0.670	−0.496	0.016^∗^	−0.745	< 0.001^∗^

^∗^ *p* < 0.05 was considered significant.

### 3.4. The Diagnostic Potential of IL‐36Ra, IL‐37, and IL‐38 in Aqueous Humor

**FIGURE 3:**
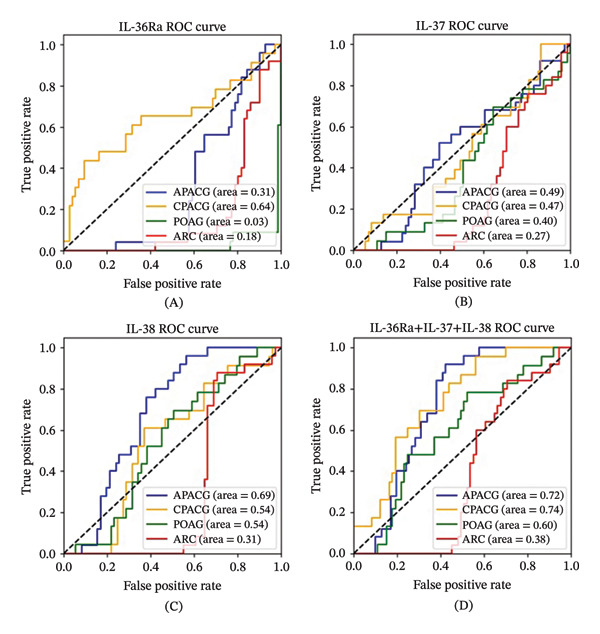
ROC curve analysis of IL‐36Ra, IL‐37, and IL‐38 for distinguishing each group from the others. (A–C) ROC curves for IL‐36Ra (A), IL‐37 (B), and IL‐38 (C). (D) ROC curve for the combined model incorporating IL‐36Ra, IL‐37, and IL‐38.

To evaluate the diagnostic utility of IL‐36Ra, IL‐37, and IL‐38 in aqueous humor, ROC curve analysis was performed for each group. Logistic regression models with 10‐fold cross‐validation were used to calculate cross‐validated area under the curve (AUC) values. In the univariate ROC analysis, IL‐36Ra showed moderate diagnostic performance for CPACG, while IL‐38 demonstrated similar utility for APACG, each achieving AUC values greater than 0.60 (Figure 3A–C). According to previous literature [28], an AUC > 0.70 is generally considered indicative of acceptable discriminatory power for individual classification. Notably, a linear combination of IL‐36Ra, IL‐37, and IL‐38 improved the predictive accuracy, yielding an AUC of 0.72 (95% CI: 0.62–0.82) for APACG and 0.74 (95% CI: 0.63–0.84) for CPACG (Figure 3D). These findings suggest that a multiplexed cytokine panel may have diagnostic value for distinguishing CPACG and APACG from other conditions.

### 3.5. Multivariable Linear Regression Analysis

To further address the potential confounding effect of disease severity, multivariable linear regression analyses were performed with adjustment for age, sex, and RNFLT, with sensitivity analyses replacing RNFLT with MDVF. Collinearity assessment showed a strong correlation between MDVF and C/D (*r* = 0.845) (Table 5), while RNFLT was moderately correlated with MDVF and C/D; therefore, these severity indicators were not entered into the same model simultaneously.

**TABLE 5:** Correlation matrix of disease severity indicators.

Variable	Average RNFLT	MDVF	C/D	IOP
Average RNFLT	1.000	−0.386	−0.493	0.233
MDVF	−0.386	1.000	0.845	0.264
C/D	−0.493	0.845	1.000	0.122
IOP	0.233	0.264	0.122	1.000

*Note:* Values are Pearson correlation coefficients.

Using ARC as the reference group, CPACG remained significantly associated with higher IL‐36Ra levels (*β* = 1.35, *p* = 0.005), whereas APACG was associated with lower IL‐38 levels (*β* = −21.81, *p* < 0.001) (Table 6). Similar results were observed in sensitivity analyses using MDVF instead of RNFLT.

**TABLE 6:** Multivariable linear regression analysis of cytokine levels.

Cytokine	Model	Variable	Coefficient	*p*
IL‐36Ra	Base (adjusted for RNFLT)	Intercept	3.119	0.013
		APACG vs. ARC	0.196	0.678
		CPACG vs. ARC	1.350	0.005^∗^
		POAG vs. ARC	0.878	0.050
		Age	0.014	0.382
		Sex (male vs. female)	0.387	0.180
		Average RNFLT	−0.003	0.497
	Sensitivity (adjusted for MDVF)	Intercept	2.815	0.018
		APACG vs. ARC	0.059	0.890
		CPACG vs. ARC	1.374	0.006^∗^
		POAG vs. ARC	0.865	0.080
		Age	0.015	0.358
		Sex (male vs. female)	0.386	0.186
		MDVF	0.002	0.928

IL‐37	Base (adjusted for RNFLT)	Intercept	16.528	< 0.001
		APACG vs. ARC	−3.097	0.051
		CPACG vs. ARC	1.989	0.202
		POAG vs. ARC	2.390	0.106
		Age	0.001	0.991
		Sex (male vs. female)	0.038	0.968
		Average RNFLT	0.018	0.287
	Sensitivity (adjusted for MDVF)	Intercept	17.832	< 0.001
		APACG vs. ARC	−2.433	0.093
		CPACG vs. ARC	1.539	0.342
		POAG vs. ARC	1.967	0.229
		Age	−0.002	0.969
		Sex (male vs. female)	−0.034	0.972
		MDVF	0.037	0.571

IL‐38	Base (adjusted for RNFLT)	Intercept	113.097	< 0.001
		APACG vs. ARC	−21.809	< 0.001^∗^
		CPACG vs. ARC	−1.998	0.704
		POAG vs. ARC	0.544	0.913
		Age	−0.364	0.045^∗^
		Sex (male vs. female)	−4.113	0.207
		Average RNFLT	−0.085	0.143
	Sensitivity (adjusted for MDVF)	Intercept	105.975	< 0.001
		APACG vs. ARC	−25.104	< 0.001^∗^
		CPACG vs. ARC	−1.019	0.854
		POAG vs. ARC	0.833	0.882
		Age	−0.347	0.059
		Sex (male vs. female)	−4.045	0.226
		MDVF	−0.013	0.952

^∗^ *p* < 0.05 was considered significant.

### 3.6. The Level of IL‐36Ra, IL‐37, and IL‐38 in the Plasma

**FIGURE 4:**
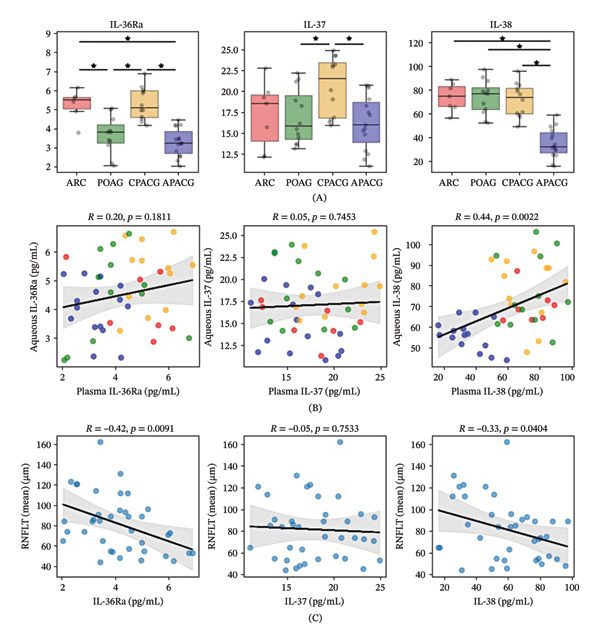
Plasma levels of IL‐36Ra, IL‐37, and IL‐38, and their correlations with aqueous humor levels and RNFLT. (A) Plasma expression levels of IL‐36Ra, IL‐37, and IL‐38 across groups. (B) Correlation between plasma and aqueous humor levels of IL‐36Ra, IL‐37, and IL‐38. (C) Correlation between plasma cytokine levels and average retinal nerve fiber layer thickness (RNFLT). ^∗^ *p* < 0.05.

Plasma samples were collected one day prior to surgery, and the concentrations of IL‐36Ra, IL‐37, and IL‐38 were measured in the corresponding subjects (Figure 4A). IL‐36Ra levels were significantly lower in the POAG and APACG groups compared to the ARC and CPACG groups. IL‐37 expression was significantly elevated in the CPACG group, while IL‐38 levels were markedly reduced in the APACG group relative to all other groups.

In addition, to assess the relationship between cytokine levels in aqueous humor and plasma, correlation analyses were conducted. A significant positive correlation was observed for IL‐38 between the two tissues (Figure 4B), suggesting its potential utility as a systemic biomarker.

Furthermore, correlations between plasma cytokine levels and average RNFLT were examined (Figure 4C). Plasma IL‐38 remained significantly negatively correlated with RNFLT (each 1 pg/mL increase in IL‐38 corresponding to an approximate 0.41 μm decrease in average RNFLT). Similarly, IL‐36Ra in plasma also showed a significant negative association with RNFLT (a 1 pg/mL increase in IL‐36Ra was associated with a 9.14 μm reduction in RNFLT). These findings further support the relevance of plasma cytokine levels, particularly IL‐38 and IL‐36Ra, in glaucomatous neurodegeneration.

## 4. Discussion

In this study, we explored the expression profiles of the anti‐inflammatory cytokines IL‐36Ra, IL‐37, and IL‐38 in both aqueous humor and plasma samples from patients with different subtypes of primary glaucoma. By systematically analyzing these cytokines across multiple glaucoma subtypes and various tissues for the first time, our study demonstrated a potential link between these cytokines and glaucomatous neurodegeneration and suggests that IL‐36Ra and IL‐38 may be a useful biomarker for distinguishing among primary glaucoma subtypes.

IL‐36Ra, a key member of the IL‐36 cytokine family, primarily exerts its biological effects through competitive binding with IL‐36 receptors. This interaction results in the inhibition of downstream signaling cascades, thereby suppressing of immune‐inflammatory responses. Previous studies have demonstrated the involvement of IL‐36Ra in the pathogenesis of several ocular diseases [29–31]. Zhao et al. [32] reported elevated IL‐36Ra expression in the aqueous humor during the active phase of uveitis, suggesting its role in dampening local inflammatory responses within the eye. Similarly, Li et al. [26] showed that IL‐36Ra exerted a protective effect against hyperosmotic stress in human corneal epithelial cells by downregulating the expression of proinflammatory mediators such as TNF‐α and IL‐1β. Despite these insights, the role of IL‐36Ra in glaucoma has remained largely unexplored. In our study, we observed significantly elevated IL‐36Ra levels in both aqueous humor and plasma samples from CPACG patients compared to POAG and APACG. ROC curve analysis indicated that aqueous humor IL‐36Ra exhibited moderate diagnostic value for CPACG (AUC = 0.64), suggesting its potential involvement in the disease’s pathophysiology. Multivariable regression analyses adjusting for disease severity (RNFLT or MDVF) confirmed that CPACG remained independently associated with higher IL‐36Ra levels (*β* = 1.35, *p* = 0.005), further supporting its potential involvement in the pathophysiology of CPACG. Unlike POAG and APACG, CPACG patients exhibit sustained angle closure, which may induce mechanical damage to the TM and disruption of the blood‐aqueous barrier, ultimately resulting in a sustained local inflammatory response with elevated cytokine expression in both intraocular and systemic compartments [33]. The observed upregulation of IL‐36Ra may reflect a compensatory anti‐inflammatory response aimed at counteracting this chronic inflammation. Nevertheless, the underlying regulatory mechanisms governing IL‐36Ra expression in CPACG require further investigation.

IL‐37, primarily secreted by monocytes and dendritic cells, exhibits anti‐inflammatory properties by effectively inhibiting the expression of proinflammatory cytokines when activated in the context of inflammation and autoimmune disorders [34, 35]. Elevated IL‐37 levels have been reported in various chronic inflammatory and autoimmune disorders, including rheumatoid arthritis, systemic lupus erythematosus, and atherosclerosis, with its expression correlated with disease severity [36, 37]. In the context of glaucoma, previous studies have identified significantly increased IL‐37 levels in the aqueous humor of patients with CPACG and POAG. Additionally, these elevated levels were found to correlate positively with the severity of VF defects, suggesting a role for IL‐37 in disease progression and optic nerve damage [25]. Our findings are in line with previous literature, as both CPACG and POAG cohorts exhibited notably higher IL‐37 levels in aqueous humor compared to the ARC and APACG groups. Notably, plasma IL‐37 concentrations in the CPACG group were markedly higher than those in APACG and POAG subjects, suggesting a chronic and long‐lasting inflammation existing in CPACG. In contrast, no statistically significant differences in IL‐37 levels were observed between APACG and ARC groups in either aqueous humor or plasma samples. However, after adjustment for disease severity (RNFLT or MDVF) in multivariable regression, these associations were no longer statistically significant (*p* > 0.05 for both CPACG and POAG). This finding indicates that the elevated IL‐37 levels observed in chronic glaucoma may be largely attributable to the extent of structural damage rather than reflecting a subtype‐specific pathophysiological feature.

IL‐38 shares homology with IL‐36Ra and exerts its anti‐inflammatory effects by competitively binding to IL‐36 receptors, thereby antagonizing the IL‐36 signaling pathway [30]. Previous research has indicated dysregulated expression of IL‐38 in various diseases such as ankylosing spondylitis, cardiovascular diseases, and rheumatoid arthritis, suggesting a broader role in modulating immune responses across multiple disease contexts [38–40]. However, currently limited research is studied on IL‐38 in the field of glaucoma. Preliminary research has reported elevated IL‐38 expression in the aqueous humor of CPACG patients, which was positively correlated with VF mean defects, suggesting an association between IL‐38 and glaucomatous damage [25]. In unadjusted analyses, aqueous humor IL‐38 levels were higher in CPACG and POAG than in ARC or APACG, with no difference between the two chronic subtypes. After multivariable adjustment for disease severity (RNFLT or MDVF), the CPACG versus ARC difference lost significance (*p* = 0.704), whereas the lower IL‐38 levels in APACG remained significant (*β* = −21.81, *p* < 0.001). Despite this loss of group difference, subgroup correlation analyses showed that IL‐38 levels were negatively correlated with average RNFLT in both CPACG (*r* = −0.496, *p* = 0.016) and POAG (*r* = −0.745, *p* < 0.001), with a stronger correlation in POAG. These findings indicate that elevated IL‐38 levels are associated with the severity of structural damage in chronic glaucoma, suggesting that the higher levels in CPACG are largely driven by damage extent rather than an intrinsic feature of this subtype. In contrast, APACG patients had significantly lower IL‐38 levels than those with CPACG or POAG, and no correlation with RNFLT was observed in this acute subtype. Notably, the higher RNFLT in APACG may reflect transient edema rather than true integrity. Unlike the chronic course of CPACG and POAG, APACG is acute, and disease duration was not analyzed, warranting caution in interpreting its correlations. In this context, the lower IL‐38 levels and absent correlation with RNFLT in APACG may be partly explained by the unique inflammatory microenvironment of acute angle‐closure glaucoma, where rapid IOP elevation triggers an explosive but transient response that may overwhelm compensatory mechanisms such as IL‐38 upregulation [41]. Collectively, these findings suggest that IL‐38 is associated with chronic glaucomatous damage in CPACG and POAG, though whether this reflects a protective response or an epiphenomenon cannot be determined from the current cross‐sectional data; further mechanistic studies are warranted.

However, several limitations should be acknowledged. First, all patients received topical IOP‐lowering medications prior to aqueous humor sampling. While this reflects routine clinical practice, the potential immunomodulatory effects of these agents on cytokine levels cannot be fully excluded. Second, the higher RNFLT observed in the APACG group may be confounded by acute optic nerve head edema, warranting caution when interpreting correlation analyses involving this subgroup. Third, the time scale of disease duration differs fundamentally between acute subtypes (measured in hours to days) and chronic subtypes (measured in months to years). Because disease duration data were not analyzed, the extent to which the observed cytokine differences between acute and chronic glaucoma subtypes can be attributed to the length of disease duration remains undetermined. To address these gaps, future studies should include larger cohorts with detailed stratification by glaucoma subtype, disease stage, and disease duration, as well as mechanistic investigations to elucidate the roles of inflammatory mediators across glaucoma subtypes.

## 5. Conclusion

In summary, this study demonstrated distinct expression patterns of IL‐36Ra, IL‐37, and IL‐38 in the aqueous humor and plasma among different subtypes of primary glaucoma. After adjustment for disease severity, elevated IL‐36Ra in CPACG and reduced IL‐38 in APACG remained independently associated with their respective subtypes, supporting their potential as subtype‐related biomarkers. A subtype‐dependent association between aqueous humor IL‐38 levels and RNFLT thinning was observed, particularly in patients with POAG and CPACG, indicating its strong association with structural glaucomatous damage. However, given the cross‐sectional design and the lack of disease duration data, further longitudinal and mechanistic studies are needed to clarify the roles of these cytokines in glaucoma pathogenesis.

## Author Contributions

Yawen Li conceived and designed the experiments. Yawen Li, Xuanqi Zhang, Xiaowei Yan, Yulei Geng, Kuitang Shi, Weijia Li, Tianyu Zhang and Jiaming Lu performed the experiments. Xuanqi Zhang analyzed the data. Yawen Li and Xuanqi Zhang wrote the original draft. Hengli Zhang and Yizhen Tang reviewed the final manuscript.

## Funding

This work was supported by the National Natural Science Foundation of China (Grant No. 82201174), Hebei Province Key Research and Development Program Project (223777110D), Beijing Natural Science Foundation (7232025), and Beijing Municipal Health Commission (QML20230203).

## Disclosure

All authors have read and approved the final manuscript for publication.

## Ethics Statement

The study was approved by the ethics committee of Shijiazhuang People’s Hospital (Approval No. 2022028; approval date: 30 March 2022).

## Conflicts of Interest

The authors declare no conflicts of interest.

## Data Availability

The research data used to support the findings of this study are available from the corresponding authors upon request.
